# Clinical and Radiographical Outcomes of Restorative Treatment With Stainless Steel Crowns Performed by Undergraduate Dental Students in Medina, the Kingdom of Saudi Arabia

**DOI:** 10.7759/cureus.49906

**Published:** 2023-12-04

**Authors:** Soha Alqadi, Omniah Aljehani, Yara E Kurdi, Mohammed Alqadi, Reem Naaman, Amnah A Algarni

**Affiliations:** 1 Preventive Dental Sciences, College of Dentistry, Taibah University, Madinah, SAU; 2 Oral Medicine, College of Dentistry, Taibah University, Madinah, SAU; 3 General Dentistry, College of Dentistry, Taibah University, Madinah, SAU; 4 Dentistry, College of Dentistry, Taibah University, Madinah, SAU; 5 Restorative Dental Sciences, College of Dentistry, Taibah University, Madinah, SAU

**Keywords:** stainless steel crown, paediatric dentistry, primary molars, dental restorations, s: dental caries

## Abstract

Background and objective

Stainless steel crown (SSC) placement has long been the preferred restorative treatment modality for treating multi-surface carious primary molars. This study aimed to assess the outcomes of SSC placement on primary molars performed by undergraduate dental students.

Materials and methods

A total of 112 children aged four to eight years were contacted for follow-ups 12 months after they underwent SSC restorations by fifth- or sixth-year female dental students in 2018-2020. Clinical and radiographic examinations were performed by trained dentists to evaluate for signs of failure. Descriptive statistics were applied for categorical variables and a Chi-squared test was used to assess the relationship between failure rate and demographic variables (i.e., age, gender, and type of tooth).

Results

The majority of the included children were aged seven years, with females (52.7%) slightly outnumbering males (47.3%). The failure rate clinically was 17.8%, involving one or more of the following signs: pain (16.1%), poor crown adaptation (16.1%), improper marginal seal (13.4%), fistula (6.3%), and mobility (4.5%). The rate of failure as per radiological examinations was 15%, involving one or more of the following signs: furcation involvement (9.8%), periapical radiolucency (3.6%), and pathological root resorption (1.8%). No significant association was found between failure rate and age, gender, or type of tooth.

Conclusions

The restorative treatment of primary molars with SSCs exhibited a high success rate when performed by less experienced undergraduate dental students from different academic levels. The failure rate did not appear to be associated with the child’s age and gender or the type of tooth.

## Introduction

Despite the increased level of awareness about oral and dental health among the general public, dental caries remains a significant health issue worldwide [1]. According to previous studies, the prevalence of dental caries is high in developing countries. For example, China, India, and South Africa have a prevalence of 85%, 53%, and 49%, respectively. These rates are higher when compared with more developed countries such as England and Italy, with prevalence rates of 32% and 16%, respectively [2-7]. In Saudi Arabia, the prevalence of dental caries is comparable to that of developing countries, with Jeddah, for example, registering a prevalence of 89% among pre-school children compared with 74.8% in Riyadh [8,9]. Reducing the level of dental caries is essential for addressing the associated effects on quality of life. These negative effects usually manifest as pain, esthetic problems, loss of appetite, sleep disturbance, and poor school attendance [10].

The treatment of dental caries varies depending on the child’s chief complaint, age, medical condition, behavioral aspects, the extent of the lesion, parents’ beliefs, and the clinical skills of the dentist [11]. Treatment modalities for the management of compromised primary teeth include either restorations or extractions and space maintenance, if needed, while restorative options for primary dentition include amalgam restoration, glass-ionomers, composite resins, and stainless steel crowns (SSCs) [11].

An SSC is defined as “a prefabricated crown form that is adapted to an individual tooth and cemented with a biocompatible luting agent” [12]. It is considered the best option in the restoration of multi-surface carious teeth [13]. The technique is used in primary and permanent posterior teeth with enamel defects such as hypoplasia, failure of previous direct restorations, coronal restoration following pulpotomy and pulpectomy procedures, high caries-risk children, and for treatment under general anesthesia [12]. In addition, retrospective studies have demonstrated that primary molars restored with SSCs after indirect pulp treatment and pulpotomy had a higher success rate compared with amalgam and composite restorations [14]. However, there is a scarcity of studies that assess the clinical outcomes of SSC procedures performed by undergraduate students or less experienced dentists. In light of this, this study aimed to investigate the success rate of SSC placement on primary molars by undergraduate dental students.

## Materials and methods

Study design and setting

In this clinical trial, the clinical and radiographical outcomes of conventional SSC procedures were evaluated one year after their placement. The procedure was performed by undergraduate dental students at Taibah University dental clinics between 2018 and 2020. The evaluation was performed by two trained dentists.

Study participants

The children's age at the time of SSC placement ranged from four to eight years and the clinical procedure involved the use of a conventional method of SSC placement on the primary molars. Any participant who did not fulfill these criteria was excluded.

Data collection technique and study outcomes

A non-probability convenient sampling method was utilized in this study. The sample was obtained from the electronic database (CS R4 electric Kodak system). The procedure had to be performed by a student in their last two years of the dental program (i.e., fifth and sixth year) at Taibah University dental clinics and supervised by a faculty member from the Pediatric Dentistry Division. Each supervisor had to examine the tooth and give approval before starting the procedure. This procedure is standardized and follows a specific protocol that has been agreed upon previously in a Pediatric Department meeting. Occlusal surfaces of the molars must be reduced by 1-1.5 mm using a 169 L tapered fissure bur on a high-speed hand-piece. The next step involves proximal reduction and breaking the contact by using the same tapered fissure bur or a thin tapered diamond bur. All line angles must be rounded and a smooth feather edge of the proximal gingival margin should be obtained. After completing the crown preparation, the appropriate size of the SSCs (3M^TM^ Unitek^TM^ Stainless Steel Primary Molars crowns) was selected by measuring the mesiodistal width of the tooth. The self-curing glass ionomer cement (Vivaglass CEM, Ivoclar, Schaan, Liechtenstein) was used by all students for crown cementation. A standardized rubric was used to evaluate students’ work after the completion of the procedure. 

After the subjects were selected and enrolled in the study, the mother of each child was contacted and given another check-up appointment 12 months after the SSC placement visit. The examiners were general dentists, and they participated in training sessions by faculty members on the clinical and radiographic evaluation methods. Clinical and radiographic (periapical radiographs) examinations were conducted during the follow-up visit to evaluate the status of the SSCs. All cases were evaluated by trained dentists. The clinical evaluation involved (1) the degree of mobility, where the tooth was held between a metallic instrument and finger and moved back and forth; (2) signs of pain and infection, such as fistula with puss discharge related to the SSC-treated tooth; and (3) loss of marginal seal and crown adaptation, such as crown mobility, loss of crown, or fluid discharge from under the crown. The radiographic variables included the presence of periapical or furcation radiolucency, pathological root resorption, and the quality of the pulp therapy in the case where the SSC was placed following therapy. Any signs of infection, mobility, improper fitting, or continuous pain following SSC placement were considered signs of failure.

Ethical approval 

Ethical approval for the study was obtained from the Institutional Review Board (IRB), College of Dentistry, Taibah University (TUCDREC/20200318/OSAlie), and the study was conducted in accordance with the guidelines of the Declaration of Helsinki (2000). The legal guardian of the child had to give consent before commencing the treatment. During the recall visit, consent was again obtained for research purposes.

Statistical analysis

The analysis was performed using SPSS® Statistics software, version 22.0 (IBM Corp., Armonk, NY). Descriptive statistics were used to obtain the frequency and percentage for all categorical variables (presence/absence of one or more signs of failure including signs of infection, mobility, improper fitting, or continuous pain following placement of SSC). A Chi-squared test was used to assess the relationship between the categorical variables. The accepted level of significance was p≤0.05.

## Results

More than half of the participants were females (52.7%) and most were aged seven years or over (74.1%) at the time of SSC placement. Lower right first primary molar (LRD) constituted 19.6% of the sample, followed by lower right second primary molar (LRE) (18.8%), lower left first primary molar (LLD) (15.2%), upper right first primary molar (URD) (14.3%), and lower left second primary molar (LLE) (13.4%) (Table 1).

**Table 1:** Demographic data of the participants URD: upper right first primary molar; URE: upper right second primary molar; ULD: upper left first primary molar; ULE: upper left second primary molar; LRD: lower right first primary molar; LRE: lower right second primary molar; LLD: lower left first primary molar; LLE: lower left second primary molar

Variable	Values (n=112)
	N (%)
Gender	
Male	53 (47.3)
Female	59 (52.7)
Age group, years	
4–5	7 (6.3)
5–6	3 (2.7)
6–7	19 (17)
>7	83 (74.1)
Tooth type	
URD	16 (14.3)
URE	8 (7.1)
ULD	8 (7.1)
ULE	5 (4.5)
LRD	22 (19.6)
LRE	21 (18.8)
LLD	17 (15.2)
LLE	15 (13.4)

After the 12-month follow-up period, the clinical success rate was found to be 82.1% and the radiographic success was 86.6%. Clinical signs of failure were found in 17.8% of the cases. The clinical evaluation revealed pain following SSC placement in 16.1% of the cases, mobility in 4.5%, fistula in 6.3%, improper marginal seal of the crowns in 13.4%, and poor crown adaptation in 16.1%. Some cases had more than one clinical sign of failure. Radiological signs of failure were reported in 13.3% of cases. The radiographic evaluation showed furcation involvement in 9.8%, periapical radiolucency in 3.6%, and pathological root resorption in 1.8%. Some cases had more than one radiographic sign of failure (Table 2).

**Table 2:** Clinical and radiographic success rate evaluations The table shows the clinical and radiographical signs analyzed in evaluating SSC procedures for primary molars. Each item was evaluated separately, and the success and failure rates were assessed. The success rate was higher than the failure rate

Variable	Yes	No
	N (%)	N (%)
Clinical evaluation (n=112)		
Pain following SSC placement	18 (16.1)	94 (83.9)
Mobility	5 (4.5)	107 (95.5)
Fistula	7 (6.3)	105 (93.8)
Improper marginal seal of the crowns (open margin)	15 (13.4)	97 (86.6)
Poor crown adaptation	18 (16.1)	94 (83.9)
Clinical signs of failure	20 (17.8)	92 (82.1)
Radiographic evaluation (n=112)		
Periapical radiolucency	4 (3.6)	108 (96.4)
Furcation involvement	11 (9.8)	101 (90.2)
Pathological root resorption	2 (1.8)	110 (98.2)
Radiographic signs of failure	15 (13.3)	97 (86.6)

Regarding the association between the success rate and the sociodemographic factors and the location of the tooth, the success rate was 47.2% in males and 40.7% in females; this difference was not significant (p=0.480). Similarly, there was no significant difference in success rate between the age groups (p>0.05). The highest success rate was found in URE (75%), followed by LRD (72.7%), while the lowest rate was found in LLE (40%). However, the results revealed no significant difference in success rate in terms of tooth type (p>0.05) (Table 3).

**Table 3:** Factors associated with success or failure rates The data in the table reveals that gender, age of the child, and the location of the primary molar have no significant effect on the success and failure rate of the SSCs SSC: stainless steel crown; OR: odds ratio; CI: confidence interval; URD: upper right first primary molar; URE: upper right second primary molar; ULD: upper left first primary molar; ULE: upper left second primary molar; LRD: lower right first primary molar; LRE: lower right second primary molar; LLD: lower left first primary molar; LLE: lower left second primary molar

Variable	Success rate	Failure rate	OR (95% CI)	P-value
	N (%)	N (%)		
Gender				
Male	25 (47.2)	28 (52.8)	0.76 (0.36–1.6)	0.48
Female	24 (40.7)	35 (59.3)		
Age group, years				
4–5	1 (14.3)	6 (85.7)	Reference	
5–6	1 (33.3)	2 (66.7)	3 (0.12–73.6)	0.50
6–7	8 (42.1)	11 (57.9)	4.3 (0.43–43.7)	0.21
7	39 (47.0)	44 (53)	5.3 (0.61–46.1)	0.13
Tooth type				
URD	10 (62.5)	6 (37.5)		0.28
URE	6 (75)	2 (25)		
ULD	4 (50)	4 (50)		
ULE	2 (40)	3 (60)		
LRD	16 (72.7)	6 (27.3)		
LRE	9 (42.9)	12 (57.1)		
LLD	10 (58.8)	7 (41.2)		
LLE	6 (40)	9 (60)		

## Discussion

According to the literature, the estimated prevalence of caries in the Medina region in Saudi Arabia is high, ranging from 57.2 to 86% in primary teeth and 67.7% in permanent teeth [15]. Due to the high prevalence of caries, the assessment of SSC placement procedures is important since SSCs are considered good restorative treatment options for carious primary molars, as they are durable compared to other restorative materials [16].

In terms of the sample distribution according to tooth site, most teeth included in this study were mandibular molars. The higher incidence of mandibular versus maxillary molars being treated with SSC aligns with the findings of another study [17]. This could be attributed to carious attacks being more likely in the mandibular posterior molars than the posterior maxillary molars [18]. In our study, the total clinical success rate was 82.1%, and the radiographic success rate was 86.6%. These findings are in agreement with those of Holan et al. who reported that 84% of pulp-treated primary molars restored with SSCs were considered successful [19]. Hutcheson et al. found that 81% of teeth treated with SSCs had no clinical or radiographic changes [20].

The judgment of failure may vary from one study to another. In this study, pain and a poor marginal seal were the main causes of clinical failure. On the other hand, in a study by Hutcheson et al. [20], gingival inflammation around the crown was the primary cause of clinical failure. Crown loss and a requirement for re-cementation was the only criterion for clinical failure in a study by Sonmez and Duruturk. The failure rate in their study was only 2.4% [21]. In our study, there were various causes of radiographic failure with furcation radiolucency being the main cause. In contrast, in another study, the calcific metamorphosis was the main cause of SSC failure one year post-treatment [20].

Higher clinical success rates than those found in our study have been reported in the literature, with two three-year follow-up studies reporting success rates of approximately 97% [22,23]. Several factors, including operator skills and the type of healthcare setting, may result in better clinical outcomes. In the present study, the clinical procedures were performed under local anesthesia by undergraduate students with varying levels of clinical skills. In the study by Roberts et al., the procedures were performed by specialist practitioners, and half of the procedures were performed under general anesthesia [23]. Similarly, in a study by Amin et al., all the procedures were performed under general anesthesia [22]. In their studies, the child’s cooperation was not considered as an important factor affecting the clinical outcome.

Previous studies have proven that SSC restorations are superior and more durable than other types of restorations, particularly for pulp-treated teeth [19,21]. Covering the tooth completely with the SSC protects the pulp against leakage. Therefore, the pulp therapy success rate was higher for teeth restored with SSCs compared with other restoration methods. In some studies, pulp inflammation was considered as the criterion of failure for restoration [24], whereas others did not differentiate between failures related to pulp inflammation and those related to restorations [25]. In the present study, the failure rate was factored in regardless of whether it pertained to pulp inflammation, restoration, or a combination of both for pulp-treated teeth. Identifying the primary cause of failure for pulp-involved teeth restoration with SSCs can be challenging.

In this study, age at the time of crown placement and tooth type influenced the failure rate. However, the sample size was too small for the findings to be statistically significant.

## Conclusions

The results of this study showed a high success rate for SSCs as a restorative treatment of primary molars, even when performed by undergraduate dental students from different academic levels. No significant association was found between the child’s age, gender, or type of tooth and the failure rate of SSCs. Therefore, SSC placement is considered a successful treatment modality to manage carious primary molars.
